# An Intact PHD Finger and PHD-BRD Interdomain Linker Are Crucial for Binding of the Chromatin Remodeler Factor TIP5 to the Histone Octamer

**DOI:** 10.3390/biom16081209

**Published:** 2026-08-19

**Authors:** Pavel Čabart

**Affiliations:** Department of Cell Nucleus Plasticity, Institute of Experimental Medicine of the Czech Academy of Sciences (IEM CAS), 142 00 Prague, Czech Republic; pavel.cabart@iem.cas.cz

**Keywords:** TIP5, PHD, linker, zinc finger, histone octamer, histone H3, binding

## Abstract

The bromodomain adjacent to zinc finger (BAZ) family protein TIP5 (transcription termination factor I (TTF-I)/interacting protein 5) contains a plant homeodomain (PHD) zinc finger module that recognizes unmodified histone H3 lysine 4. This study demonstrates that the immobilized PHD domain recruits the histone octamer complex. Depletion of zinc cations from the finger or disruption via mutagenesis completely abolished this interaction. Interestingly, the binding ability of the depleted protein was partially recovered under high concentrations of KCl. Extending the PHD domain with a PHD-bromodomain (BRD) interdomain linker led to a substantial increase in binding affinity, with the magnitude progressively dependent on the linker length. To gain insight into these preferential binding interfaces, AlphaFold 3 structure predictions were performed. Within the histone octamer complex, histone H3 was identified as a primary, but not sole, binding partner for TIP5 partial proteins. An increase in predicted total van der Waals interactions correlated with the presence of the linker and its extension; however, an anomaly stemming from the calculated stickiness of the short linker version was encountered. Increased hydrogen-bonding in models with the short linker mirrored the observed affinity. Conversely, the long linker reduced predicted hydrogen (H)-bonds below that for the PHD alone. Finally, structural analysis of the disrupted zinc finger motif revealed the fewest hydrogen bonds.

## 1. Introduction

TIP5/Bromodomain adjacent to zinc finger 2A (BAZ2A) is a 205 kD nucleolar protein that is tightly associated with the catalytic subunit SMARCA5 (SWI/SNF-related, matrix-associated, actin-dependent regulator of chromatin, subfamily A, member 5), thereby forming a nucleolar chromatin remodeling complex (NoRC) [1]. TIP5 (transcription termination factor I (TTF-I)/interacting protein 5), like other members of the BAZ family of proteins, is characterized by a plant homeodomain-bromodomain (PHD-BRD) tandem module located at its C-terminus. Biochemical and structural studies identified the unmodified N-terminal tail of core histone H3 as the preferred binding partner for the TIP5 PHD domain. Histone H3 is one of the subunits of the histone octamer, which consists of 2 copies of each core histones H2A, H2B, H3 and H4 [2]. The histone octamer is located at the center of the nucleosome core particle [3]. The PHD finger recognizes the histone H3 lysine 4 (H3K4) N-terminal region of H3 through backbone hydrogen-bond interactions [4]. Earlier in vitro studies revealed that all four core histones bind to the PHD fingers 1 and 2 of the TIP5-related protein ACF1 (ATP-dependent chromatin assembly factor subunit 1) with high salt resistance. On the contrary, the association of histones with the BRD domain did not withstand the same ionic strength level [5]. Based on crystallographic studies, human TIP5 and TIP5-like protein BAZ2B have the canonical PHD finger, cross-braced folding topology with two zinc(II)-binding sites coordinated by the Cys4-His-Cys3 (C4HC3) motif [4].

TIP5 PHD finger adopts a right-handed 3_10_ helix structure [4], whereas the interdomain linker, connecting PHD and BRD, is largely disordered. Its role in mediating H3 binding to the PHD finger is still unclear. At present, two different mechanisms are proposed. Both are based on a particular abundance of acidic and basic residues in the PHD domain(s) and linker, respectively. The first model postulates that the positively charged, histone-mimicking BAZ2B linker competes with the positively charged N-terminus of H3 for binding to the negatively charged PHD [6]. A second notion stems from interactions between H3 peptides and the ATPase/helicase subunit CHD4 (chromodomain-helicase-DNA-binding protein 4), a part of the chromatin remodeler NuRD (nucleosome remodeling and deacetylase) [7]. In this case, the PHD domains (1+2) and the linker connecting them are negatively charged. To examine the role of the linker, the PHD1-linker and linker-PHD2 constructs were tested. Both linker-bearing constructs possessed a substantially higher histone H3-binding affinity than the corresponding PHD domains alone [8]. These authors hypothesize that the highly negatively charged linker can increase the local concentration of the positively charged H3 peptide, which enhances binding of the PHD fingers.

The present work demonstrates the importance of intact Zn^2+^ ion-coordinating C4HC3 architecture and PHD-BRD interdomain linker in mediating binding between the TIP5 and histone octamer. Furthermore, I find a direct correlation between increased linker length and higher histone octamer-binding affinity. Intriguingly, high ionic strength conditions counteracted the structural defect caused by Zn^2+^ deficiency. AlphaFold 3 [9] structural predictions suggest that, in addition to histone H3, other histone octamer subunits are involved in interactions that are mediated by residues within the interdomain linker.

## 2. Materials and Methods

### 2.1. Expression and Purification of Recombinant Proteins

**Full-length murine TIP5.** Baculovirus expressing His_6_-myc-tagged TIP5 [10] was amplified three times in Sf9 cells to produce high titer stock for protein expression. After each round of amplification, suitable virus concentrations were assessed using cell enlargement and SDS-PAGE analysis to check protein levels. For large scale expression, 2.2 × 10^7^ Sf9 cells/20 cm plate were seeded and allowed to attach in 10 mL of media. A P3 stock (passage 3 viral stock) was then diluted to a final ratio of 1:200 in this medium. Sixty hours after infection, a cell extract was prepared by three freeze/thaw cycles followed by three (30 s; amplitude 50%) sonication bursts in a modified EX-500 buffer [10] [500 mM KCl, 10 mM Tris-HCl (pH 7.6), 1.5 mM MgCl_2_, 10% glycerol, 1 mM β-mercaptoethanol, and protease inhibitors] containing 0.05% NP40. The extract was clarified by centrifugation at 10,000× *g* (4 °C, 30 min) and loaded on the TALON Superflow column (GE Healthcare, Chicago, IL, USA). The resin was washed with EX-500 containing 5 mM imidazole. Then, bound TIP5 was eluted with EX-500 containing 250 mM imidazole and dialyzed against EX-300 buffer (300 mM KCl).

**Partial TIP5 proteins.** All sub-fragments of TIP5 were cloned into the BamHI/NotI sites of the vector pGEX-4T3. The double mutant C1683S, H1688L was generated by PCR-based site-directed mutagenesis. The GST-TIP5 fusion proteins were expressed in *E. coli* DH5α cells. For induction, a 50 mL culture was grown at 37 °C until an OD_600_ of 0.5, induced with 0.1 mM IPTG, and incubated for an additional 3 h. Induced cells were disrupted in NETN lysis buffer [500 mM NaCl, 20 mM Tris-HCl (pH 8.0), 0.5% NP-40, 1 mM EDTA, 0.5 mM dithiothreitol (DTT), and protease inhibitors] by sonication. Clarified extract was incubated with 0.4 mL glutathione-agarose (Sigma-Aldrich, St. Louis, MO, USA) beads pre-equilibrated in NETN with 0.5% non-fat dry milk for 3 h at 4 °C. After sequential washes with NETN (500 mM, 250 mM, and 100 mM NaCl), proteins were eluted with 50 mM reduced glutathione for 1 h at 4 °C, followed by 10 min at 22 °C and then dialyzed against storage buffer (100 mM KCl, 20 mM Tris-HCl, pH 7.9, 1.5 mM MgCl_2_, 0.5 mM EGTA, 10% glycerol, 0.5 mM DTT, and 0.2 mM phenylmethylsulfonyl fluoride (PMSF)). Prior to the protein–protein interaction assays, GST and GST-TIP5 proteins were bound to glutathione-agarose beads so that they were adjusted to comparable amounts of their full-length forms.

***Xenopus laevis* histone octamer.** Polycistronic co-expression and purification of core histones H2A, H2B, H3 and H4 was carried out as described previously [11], with some modifications. Expression plasmid pET29a-YS14 [11] was transformed into BL21-CodonPlus(DE3)-RIPL strain. For induction, a 500 mL culture was grown at 37 °C until an OD_600_ of 0.4, induced with 0.4 mM IPTG, and incubated for 20 h. Collected cells were disrupted in lysis buffer (2 M NaCl, 20 mM Tris-HCl, pH 8.0, EDTA-free protease inhibitor cocktail, 1 mM β-mercaptoethanol, 1 mM PMSF) by sonication. The clarified extract was subjected to cobalt affinity chromatography, followed by step elution with 170 mM imidazole. Eluate was concentrated on an Amicon Ultra-15 centrifugal filter device (cut-off 10 kDa), and the histone concentration was determined by a Pierce BCA protein assay using bovine serum albumin (BSA) as a standard. Polyhistidine tags were removed from H2A and H4 histones using thrombin (Sigma-Aldrich) digestion overnight at 4 °C. A histone-to-thrombin mass ratio of 25:1 was used. The cleaved histidine tags and uncleaved fusion proteins were separated by passage through a cobalt column. The thrombin was removed from the histone mixture using heparin-agarose (Sigma-Aldrich) resin. Digested histones were dialyzed against 0.5 M NaCl, 20 mM Tris-HCl (pH 8.0), 1 mM β-mercaptoethanol and 0.2 mM PMSF, and their concentration was determined again. The formation and intactness of the histone octamer were verified by blue-native (BN) 4–15% polyacrylamide gel electrophoresis (PAGE), where the complex migrated as a single band at an apparent molecular weight of ~150 kDa.

### 2.2. Protein–Protein Interaction Assays

**Figure 1:**
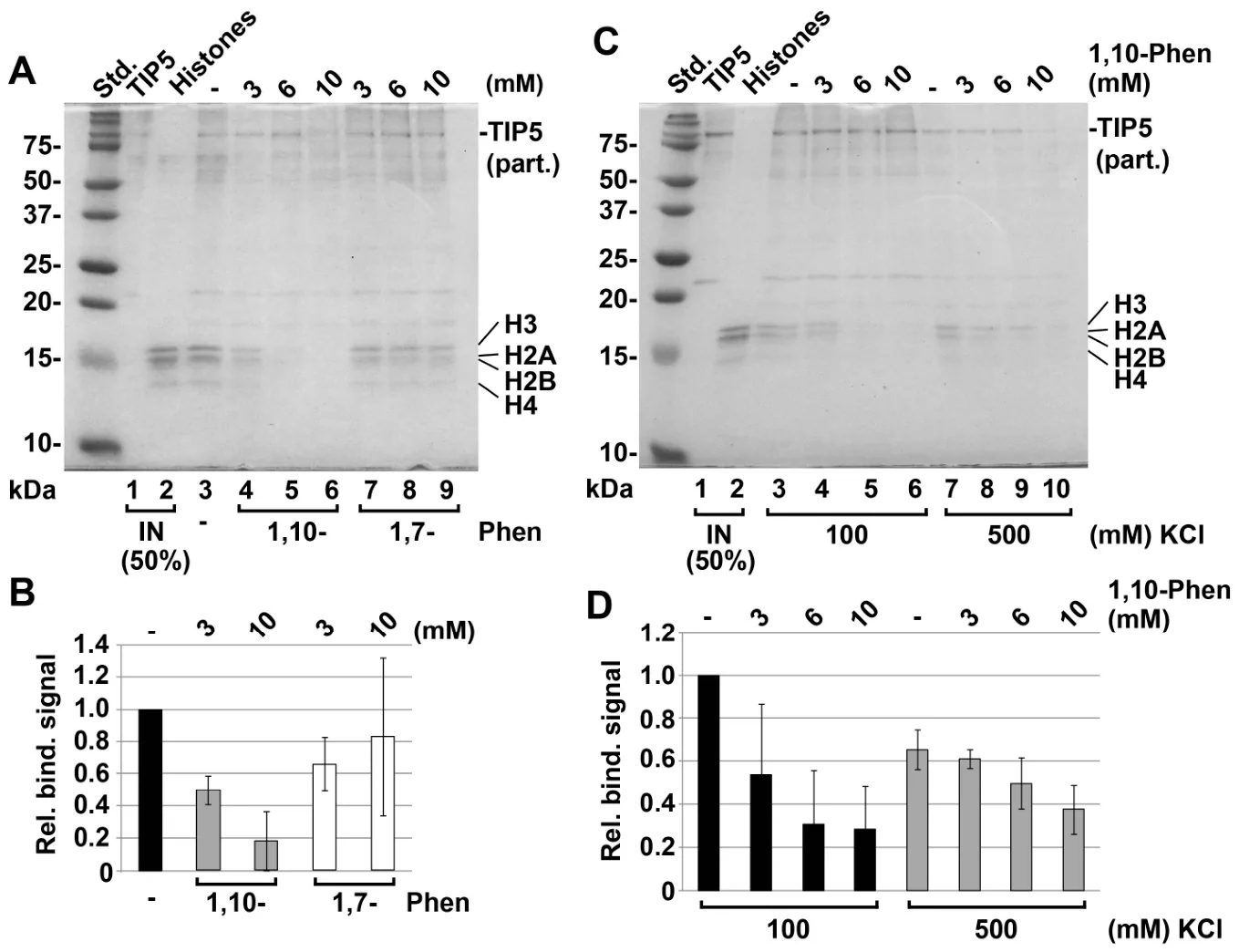
High ionic strength alleviates the metal cofactor deficiency in the contacts between TIP5 and core histones. (**A**) Baculovirus-expressed His_6_-myc-tagged recombinant TIP5 was immobilized on Co^2+^ agarose beads (via its N-terminus) and used for ‘pull-down’ assays. Purified bacteria-expressed histone octamer was incubated with the bead-bound TIP5, which had been pre-treated with buffer alone (-) or indicated concentrations of 1,10- or 1,7-Phenanthroline (Phen). The beads were extensively washed (see Materials and Methods) and the histones that remained bound were analyzed by 15% SDS-PAGE. (**B**) Histone protein levels in (**A**) were quantified and plotted relative to the level in the no-Phen control reaction ((**A**), lane 3), which was set to 1. Error bars indicate the range of values (*n* = 2). Values for reactions with 3 mM and 10 mM Phen are shown. (**C**) Following the experimental setup in (**A**), TIP5 was pre-treated with 1,10-Phen only. Buffers containing 100 or 500 mM KCl were used for octamer binding and bead washing. (**D**) Histone protein levels in (**C**) were quantified and plotted relative to the no-Phen control reaction at 100 mM KCl ((**C**), lane 3) set to 1. Error bars represent SD (*n* = 3). Panels (**A**,**B**), lanes 1 and 2 show 50% of the relevant protein input. Full-length TIP5 is absent on these gels, where only the partial protein is marked (part. TIP5).

PolyHis-TIP5, GST-TIP5 and GST proteins, bound to TALON and glutathione-agarose beads, respectively, were rotated with 1.5 μg of core histones in binding EX-300 buffer (300 mM KCl) containing 0.5% NP40 at 4 °C for 3 h. In the experiment shown in Figure 1C the binding EX buffers contained 100 mM or 500 mM KCl. Where indicated, polyHis-TIP5 and core histones were pre-treated with 1,10- or 1,7-Phenanthroline for 2 h at 30 °C, and GST/GST-TIP5 binding reactions were supplemented with 10 μM ZnCl_2_. The beads were washed four times with appropriate binding buffer, boiled in SDS sample buffer, and then pelleted; the supernatant was loaded onto an SDS-15% polyacrylamide protein gel. Proteins were detected by Coomassie Blue or silver staining and quantified using ImageJ software, v2.15.1 (Fiji). Immunoblot detection of histone octamer with the anti-histone H3 rabbit polyclonal antibody (Abcam, ab1791, Cambridge, UK) was performed using the enhanced chemiluminescence system (Thermo Scientific, Waltham, MA, USA) as recommended by the manufacturer. Levels of histone H3 were quantified using ImageJ.

### 2.3. Protein Structure Predictions

Computational modeling of three-dimensional (3D) protein structures was performed using the AlphaFold 3 artificial intelligence model [9]. Visualization of the protein structures and calculation of interaction parameters were carried out using the UCSF ChimeraX software, v1.12 [12].

**Accession Codes (UniProtKB):**

- TIP5: Q6Q074
- Histone H2A: Q6AZJ8
- Histone H2B: Q92130
- Histone H3: A0A310TTQ1
- Histone H4: P62799

## 3. Results and Discussion

### 3.1. TIP5 Forms a Complex with the Histone Octamer in a Metal-Cofactor-Dependent Fashion

It has been reported that PHD finger and BRD domain structures of the TIP5-like protein ACF1 interact with both endogenous and unmodified recombinant core histones [5]. Firstly, in order to examine if an intact TIP5 is able to interact with core histones in my in vitro pull-down assay, full-length, polyhistidine-tagged TIP5 was expressed in a baculovirus system, isolated, and immobilized on a Co^2+^-charged support through its N-terminus. This bio-affinity resin was incubated with purified recombinant histone octamer (2x [H2A, H2B, H3 and H4]) and subsequently subjected to stringent washings. Bound proteins were resolved by SDS-PAGE and visualized by Coomassie blue staining. Lane 3 in Figure 1A shows that all four histones were in complex with TIP5.

Previously, it has been found that interactions between ACF1 PHD domains and core histones are sensitive to 1,10-phenanthroline (Phen) [5]. I therefore used this chelating agent to study the possible role of the TIP5 PHD finger in making direct contacts with the histone octamer. Pretreatment of TIP5 (and histones) with increasing concentrations of 1,10-Phen was carried out before binding assays. Lanes 4–6 (Figure 1A) show a dose-dependent decrease in interaction, ultimately reaching about 5-fold inhibition at 10 mM 1,10-Phen (see quantitation in Figure 1B). To ensure the observed effect depended on metal chelating activity, the non-chelating isomer 1,7-phenanthroline was used as a control at equivalent concentrations (Figure 1A, lanes 7–9, Figure 1B). As expected, binding of TIP5 to core histones is abolished by metal removal, presumably from the zinc finger-containing PHD domain. Since only low residual interactions were detected after chelation, it can be concluded that the involvement of the BRD module is minimal to none.

### 3.2. High Ionic Strength Restores the TIP5 Interaction with Core Histones That Was Lost Due to Metal Cofactor Deficiency

It is well established that zinc finger disruption can lead to protein unfolding and loss of protein activity ([13] and references therein). Ionic-strength-dependent effects in protein folding have also been reported [14]. To investigate a possible mutual relationship between these types of perturbation, a follow-up assay was performed with the 1,10-Phen-treated TIP5 in the presence of two different salt concentrations. Because 300 mM KCl was used in the experiment in Figure 1A, lower and higher salt concentrations were tested (100 mM and 500 mM, respectively). Results from 100 mM KCl conditions (Figure 1C, lanes 4–6) are similar to those at 300 mM KCl (Figure 1A, lanes 4–6). Surprisingly, histones were still detected in parallel 500 mM KCl-reactions (compare lanes 9 and 10 with lanes 5 and 6 in Figure 1C). Quantitation of binding signals from three independent experiments (Figure 1D) exhibits steeper and shallower gradients of weakening of the binding capacity at 100 and 500 mM KCl, respectively. This is an unexpected observation, suggesting that improper folding of metal-depleted TIP5 is alleviated by higher ionic strength, which may lead to the partially restored ability of the PHD finger to bind the core histones. Further, it is reasonable to assume that the PHD finger is primarily involved in these interactions, since the ACF1 BRD module-histone interactions were not resistant to 500 mM salt conditions [5].

### 3.3. Extending the Interdomain Linker Progressively Enhances the C4HC3 Motif-Dependent Association of the TIP5 PHD Finger with the Histone Octamer

**Figure 2:**
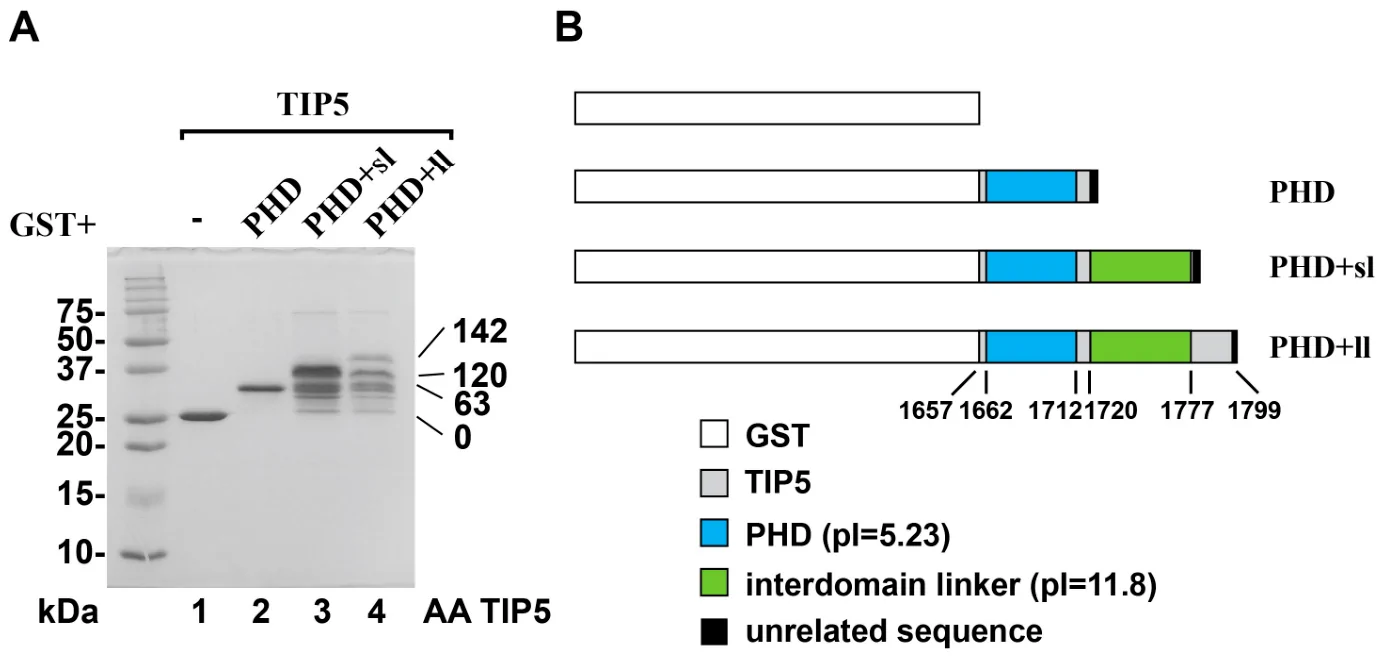
Preparation of GST-TIP5 fusion proteins for ‘pull-down’ experiments. (**A**) Indicated domains of TIP5 were expressed as GST fusion proteins and isolated on glutathione-agarose. A representative gel pattern of immobilized proteins is shown. PHD, plant homeodomain; sl, ‘short‘ linker; ll, ‘long‘ linker. On the right, the total numbers of TIP5 amino acid (AA) residues fused to the GST protein are indicated. (**B**) Schematic of partial TIP5 sequences merged with the GST moiety. Domain organization and relevant isoelectric points (pI) are shown.

It was proposed that the positively charged PHD-BRD interdomain linker in the BAZ2B protein acts as a damper of H3 histone binding by interacting with the negatively charged PHD domain [6]. This concept was derived from isothermal titration calorimetry data analysis of interactions between the N-terminal H3 histone peptide and PHD or PHD-linker regions revealing that peptide binding to PHD was by almost one order of magnitude stronger [6]. To explore this further with the full-length H3 histone in the context of the histone octamer, a set of GST-TIP5 partial mutants was designed (Figure 2B), containing only the acidic (pI = 5.23) PHD region, extended by four unrelated amino acids, Ala-Ala-Ala-Ser (denoted as PHD) and two versions of PHD-interdomain linker fusions.

The first version contains the linker per se (‘short‘ linker; sl) and is also extended by the AAAS sequence (PHD+sl). In the second version, the linker is flanked by twenty-one TIP5-specific amino acids (‘long‘ linker; ll) and three unrelated amino acids, AAS (PHD+ll) on the C-terminus. Similar to BAZ2B, the TIP5′s interdomain linker is basic (pI = 11.8). GST-fusion proteins were generated in *E. coli* and affinity purified. Panel A in Figure 2 shows a representative pattern, which served to adjust equal amounts of genuine species for input into pull-down experiments. Specifically, only top protein bands were considered, which correspond to the total numbers of TIP5 amino acids fused to GST moiety.

**Figure 3:**
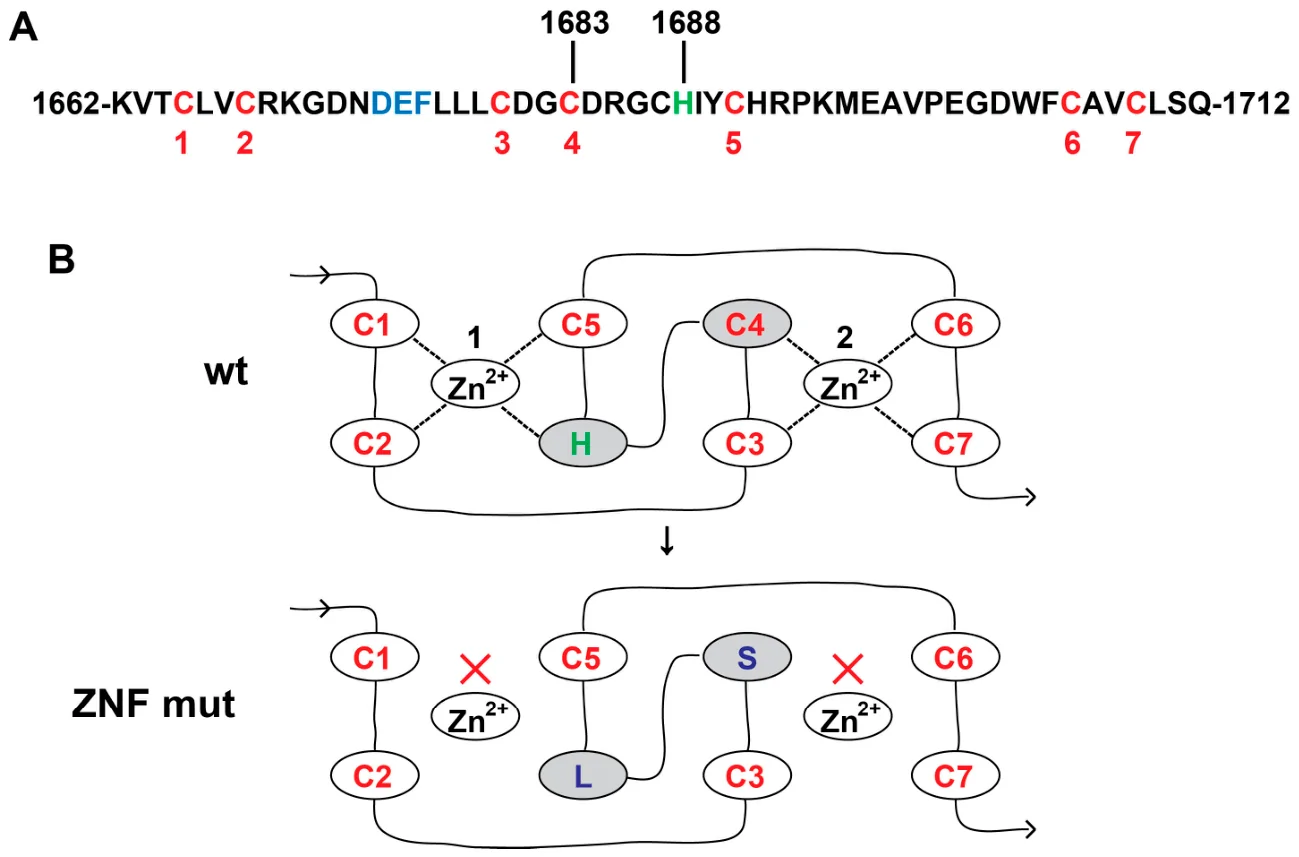
Sequence-structure organization of the PHD zinc finger and its mutation. (**A**) Residues involved in the Cys4-His-Cys3 motif and residues Asp1635, Glu1636, and Phe1637, forming the H3-binding surface, are indicated in red (C), green (H) and blue (D, E, F), respectively. (**B**) Upper part, depiction of the PHD finger ‘cross-braced’ topology with two zinc-binding sites (wt). Lower part, residues Cys1683 and His1688 were mutated to Ser and Leu, respectively, to potentially disrupt the coordination of both zinc ions (ZNF mut).

Within the Cys4-His-Cys3 motif, residues Asp1688, Glu1689 and Phe1690 were defined to form a site for H3 histone binding [4]. Corresponding residues Asp1674, Glu1675 and Phe1676 in mouse TIP5 are indicated between the second and third cysteines of the C4HC3 motif (Figure 3A).

**Figure 4:**
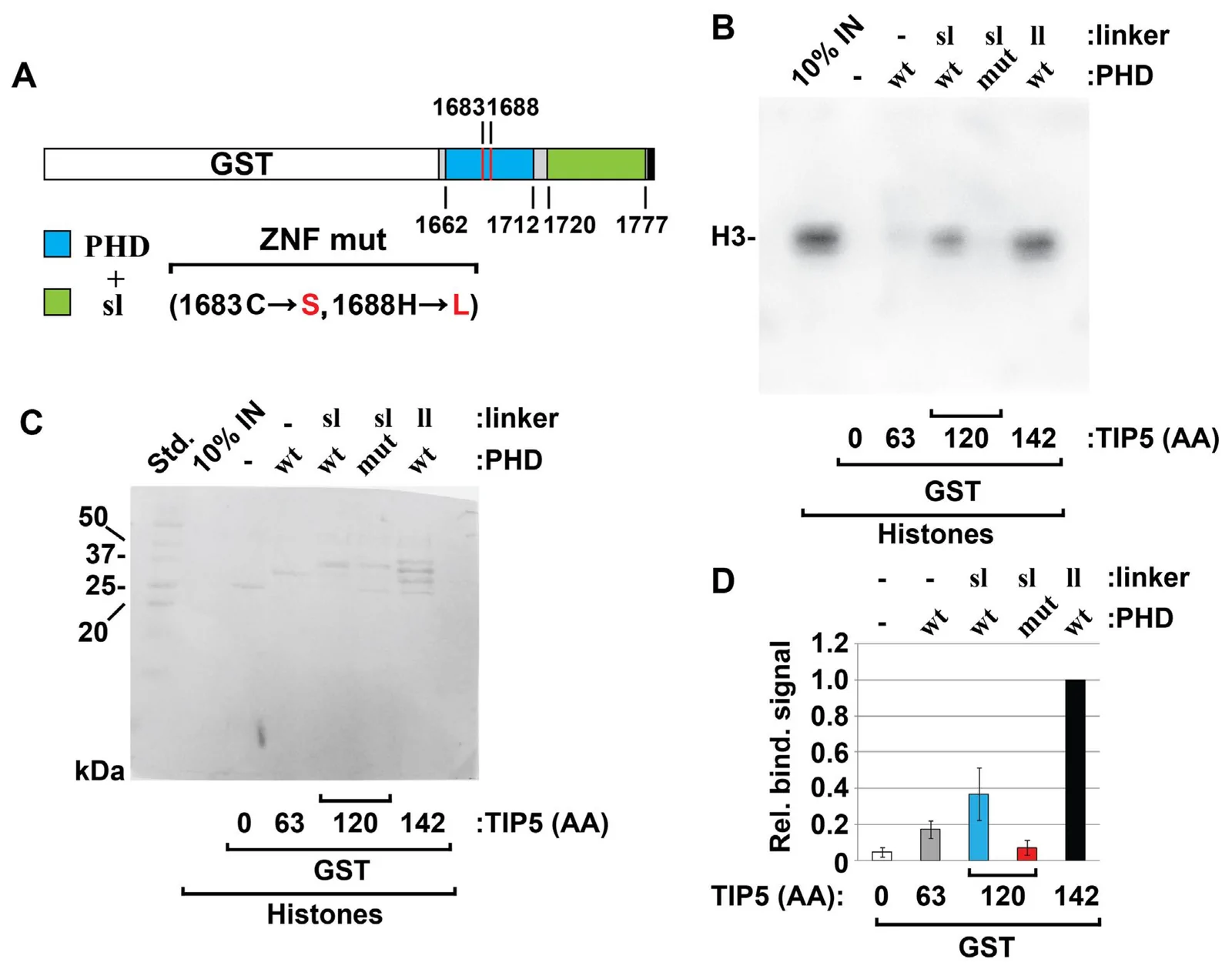
Both the wild-type PHD zinc finger motif and the interdomain linker are required for efficient binding of the PHD domain to the histone octamer. (**A**) A scheme of the GST-TIP5 zinc finger mutant. For other GST-TIP5 proteins tested, see Figure 2B. (**B**) A GST-pull down assay was performed with purified recombinant histone octamer in the presence of 10 μM ZnCl_2_. The bound histone H3 was detected by Western blotting using an anti-histone H3 antibody. The first lane shows an H3 signal originating from 10% input of the core histones. (**C**) The same blot shows Coomassie Blue-stained GST and GST-TIP5 proteins used in the binding assay. (**D**) Histone H3 levels in panel (**B**) were quantified and plotted relative to the long-linker (ll) wild-type (wt) reaction, normalized to 1. Error bars represent SD (*n* = 3).

In an attempt to disrupt both zinc ion-binding sites simultaneously, the fourth cysteine (C1683) and histidine (H1688) were substituted with serine and leucine, respectively (Figure 3B). This double mutant (see scheme on Figure 4A) was prepared in essentially the same way as the aforementioned wild-type GST-TIP5 fusions.

Subsequently, all proteins were re-bound to glutathione resin. Consistent with experiments on full-length TIP5 (Figure 1A), incubation with core histones was carried out at 300 mM KCl. Since crystallization of the *E.coli* expressed TIP5 PHD finger in its apo form was performed in a buffer containing a low concentration of ZnCl_2_ (20 μM) [4], the binding buffer was supplemented with 10 μM ZnCl_2_ to facilitate proper zinc finger folding. After stringent washing, the quantity of the bound octameric histone core was determined by detecting its H3 subunit. A typical binding pattern is shown in Figure 4B; quantification of binding signals from three independent experiments is shown in Figure 4D. The intact PHD finger domain itself (PHD wt) recruited only a small amount of octamer-complexed H3 while the inclusion of linker (PHD wt+sl) led to a stronger octamer binding. This was even more enhanced by a longer flanking sequence at the linker’s C-terminus (PHD wt+ll). Mutation of the C4HC3 motif (PHD mut+sl) caused a strong disruption of the TIP5–octamer interaction. Following blot development, the membrane was stained with Coomassie Blue to demonstrate that comparable amounts of the full-length GST-TIP5 proteins were used in binding reactions (Figure 4C).

Collectively, my data with the histone octamer are not in accordance with the finding that the basic BAZ2B PHD-BRD interdomain linker reduces the binding affinity of the PHD finger for the H3 histone [6]. On the contrary, I find the presence of a positively charged linker assists the TIP5 PHD finger domain in binding to the octamer, and that its C-terminal extension further enhances this interaction.

### 3.4. The Intact TIP5 PHD Finger Domain Likely Associates with the Full Histone Octamer

**Figure 5:**
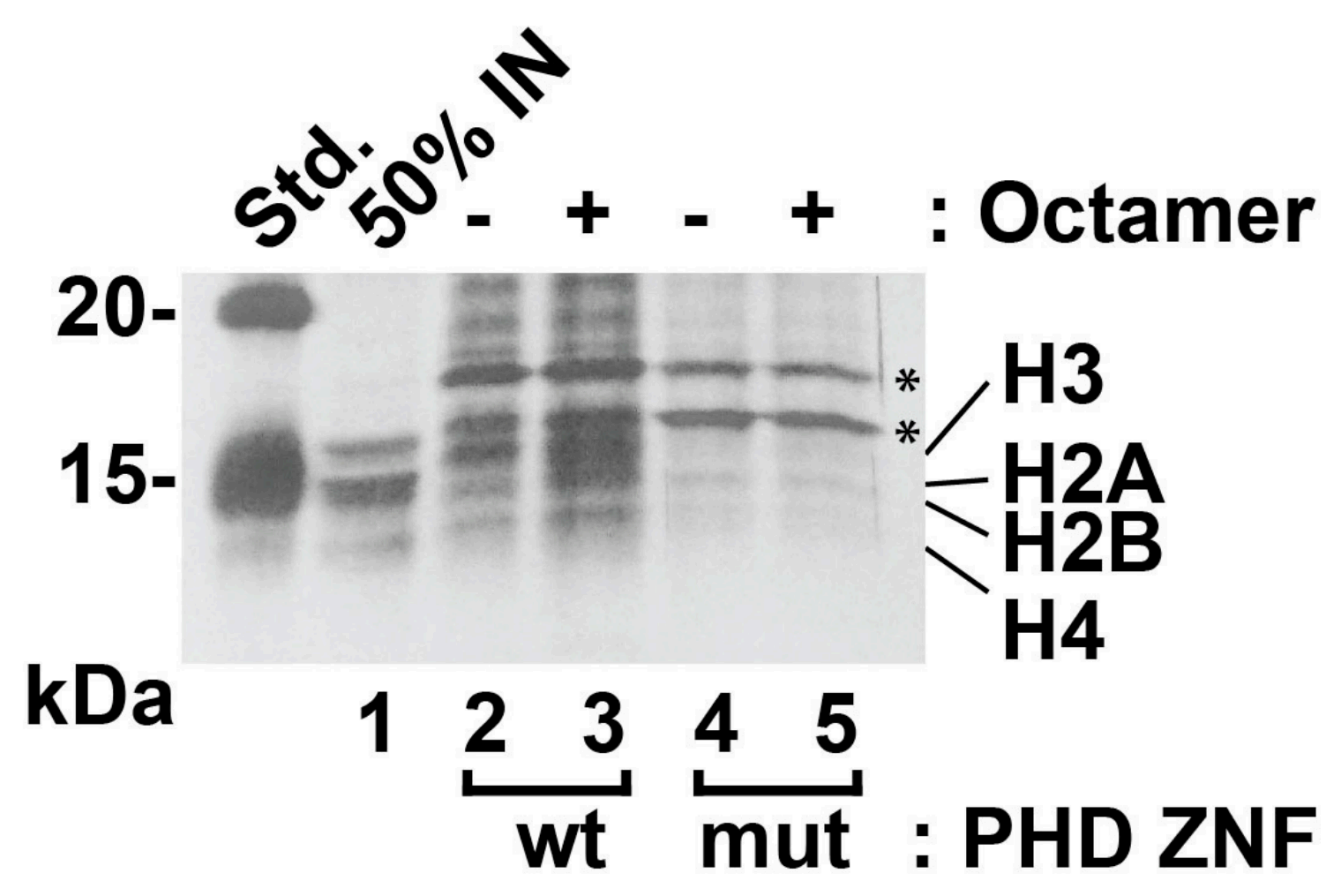
The histone octamer likely forms a complex with the intact TIP5 PHD finger domain. A pull-down assay was performed with GST-PHD+sl bearing wt (lanes 2 and 3) or mutated zinc finger (lanes 4 and 5), and histone octamer (lanes 3 and 5), in the presence of 10 μM ZnCl_2_ (lanes 2–5). Lanes 2 and 4 serve as negative controls, i.e., no histones added to the reactions. Following 15% SDS-PAGE separation, all binding partners were visualized with silver staining. Lane 1 shows 50% of the histones input. Asterisks indicate the most prominent partial GST-TIP5 products.

In line with the experiments in Figure 1, it was desired to directly demonstrate that the complete histone octamer forms a complex with the wild-type TIP5 PHD finger domain. In this pull-down assay, the resin-attached GST-TIP5 fragments consisting of the wild type or zinc finger mutated PHD domain, extended by a ‘short‘ linker were used. Beads were split into two halves; the first half was mixed with histone octamers and the second half served for pattern comparison after silver staining of resolved proteins. This was necessary to account for interfering bands of degraded or incomplete GST-TIP5 proteins. As shown in Figure 5, an enrichment of the signal in the region of interest (compare lanes 2 and 3) indicates that all four core histones appear to be present in a complex with the C-terminal extended wt PHD domain. No difference in protein pattern was observed in corresponding reactions with the mutated zinc finger (Figure 5, see lanes 4 and 5).

My data are in agreement with the pull-down experiments of Eberharter et al. [5], where a partial ACF1 structure containing PHD 1–2 and BRD domains associated with all four input histones, whether supplied as a purified endogenous histone octamer or as an octamer reconstituted from separate recombinant proteins. Association between TIP5 and histone octamer was also predictably dependent upon proper folding of zinc finger domain (Figure 1A,B). However, the presence of histones in complexes with the zinc-deficient TIP5 in a high-salt environment was not anticipated (Figure 1B). One possible explanation is that the high concentration of salt ions (500 mM) may screen the unfavorable interactions. If there are electrostatically unfavorable interactions in the absence of zinc cations, these could be moderately mitigated by high ionic strength. Another possibility is that some potassium ions might act like zinc ions, even though they can only weakly mimic them. Yet, substitution of the Zn^2+^ for K^+^ may occur at high KCl concentrations.

**Figure 6:**
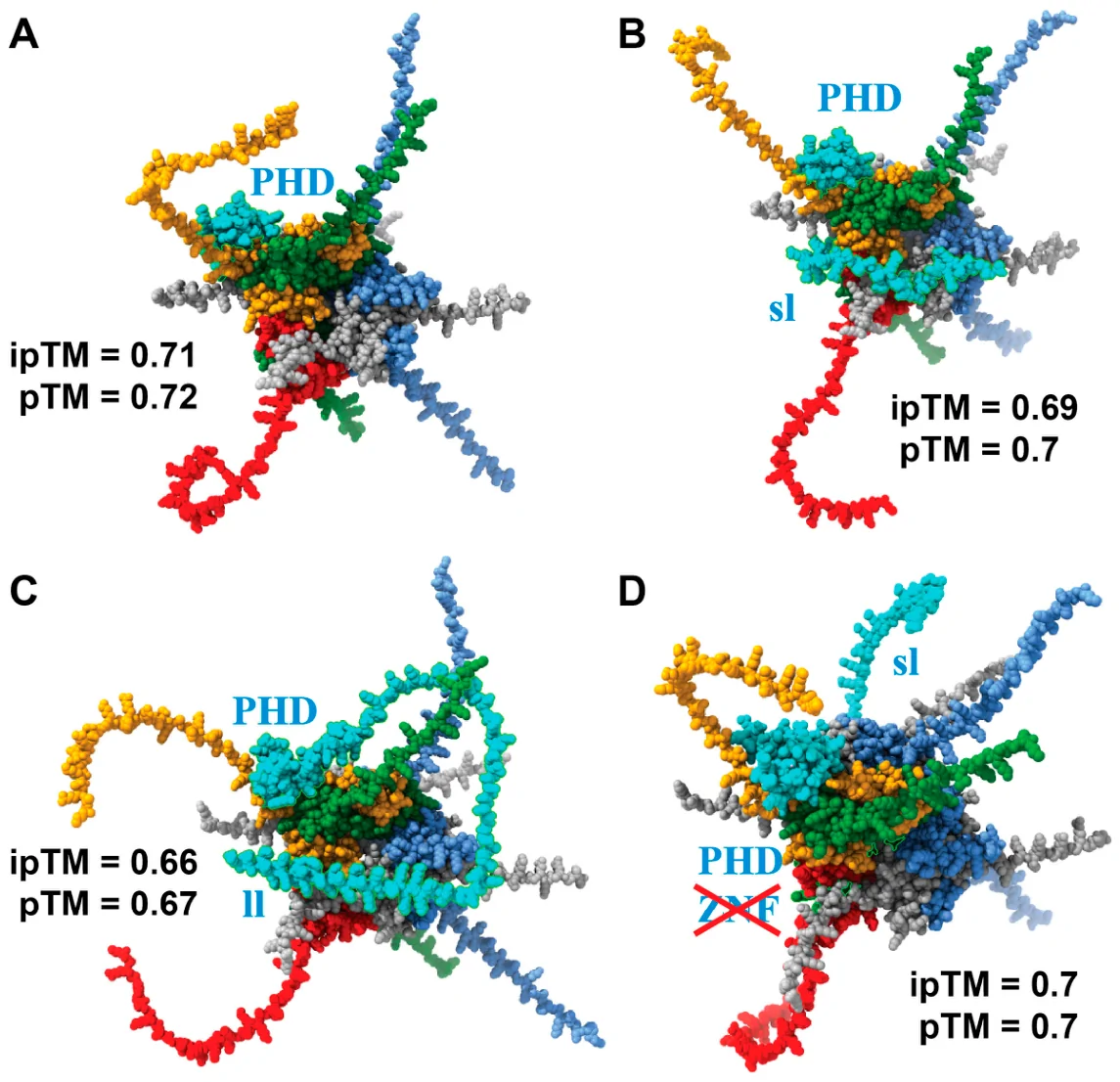
Predicted 3D structures of the TIP5 PHD domain and its extended interdomain linker fragments, bound to the histone octamer. AlphaFold 3 model of the (**A**) PHD domain alone (PHD), (**B**) PHD domain with the ’short’ linker (PHD sl), (**C**) PHD domain with the ’long’ linker (PHD ll), (**D**) PHD domain with the ’short’ linker, zinc finger mutated (PHD sl 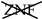). All structures are portrayed in sphere style; TIP5 partial proteins are displayed in cyan, H2A subunits in gray, H2B subunits in blue, the H3 subunit interacting with TIP5 protein in orange, the second H3 copy in red, and H4 subunits in green. Structural confidence and accuracy metrics scores, pTM and ipTM, are shown for each model.

In order to find a plausible explanation for the increasing strength of association between the histone octamer and the PHD module, extended by the ‘short‘ and ‘long‘ versions of the interdomain linker, all relevant sequences and two Zn^2+^ cofactors were submitted to the AlphaFold 3 server for three-dimensional (3D) structure predictions. The GST tag sequence was omitted because it affects structural predictions. Out of five models obtained for each complex, the most representative structures were selected based on having the highest predicted template modeling (pTM) [15] and interface predicted template modeling (ipTM) [16] scores (Figure 6A–D).

Primary binding partner for the PHD module (shown in cyan) was histone H3 (depicted in orange). For simplicity, models with one molecule of the TIP5 protein fragment are presented. Another TIP5 fragment was predicted to associate, in a symmetrical way, predominantly with the second copy of histone H3 (depicted in red; Supplemental Material, Figure S1). The question is whether it is physiologically relevant due to potential steric hindrance. Due to its large size, two copies of full-length TIP5 may not be simultaneously accommodated. Moreover, TIP5 forms a complex with SMARCA5 and other factors, with an estimated native molecular weight of >800 kDa [1,17]. AlphaFold has predicted interactions between the PHD and H3 despite the mutated zinc finger motif (Figure 6D); however, see Table 1 for further analysis. The interdomain linker exhibits the expected flexibility. Depending on the length, it makes multiple contacts with other subunits of the histone octamer (Figure 6B; PHD sl; Table 1) or forms loops that make fewer contacts with the octamer body (Figure 6C; PHD ll; Table 1).

To obtain an unbiased overview of putative interaction modes, four parameters were retrieved from all five models and summed for each complex (Table 1). Since the structural confidence and accuracy scores for pTM [15], and ipTM [16] ranged from ~0.66 to ~0.72, information derived from these values should be considered exploratory and model-dependent.

In the upper section, the total number of predicted interatomic contacts differs significantly between the PHD and PHD+sl models, with latter showing an almost 3-fold increase. On the other hand, the number of octamer contacts with PHD+ll drops to 83.6% relative to PHD. The PHD+sl ZNFmut version retains the high contacting properties of its wt counterpart, while a nearly 20% decrease can be attributed to a misfolded zinc finger. It needs to be emphasized that the raw number of contacts between two binding partners describes the size of their physical interface rather than directly measuring their binding affinity. Further, two types of intermolecular electrostatic forces critical for protein–protein interactions were the focus: van der Waals (vdW) forces and hydrogen bonds (H-bonds).

In a large number of protein complexes, vdW forces can constitute approximately 75% of the total binding energy, while specificity and directionality are governed by H-bonds, which contribute roughly 15% of total stabilization energy [18]. Remarkably, there were more than 12- and about 10-fold increases in the number of vdWs for PHD+sl and PHD+sl ZNFmut, respectively, but only ~1.4-fold for PHD+ll. Regarding the H-bonds, a total of 42 bonds were identified for PHD alone, the ‘short‘ linker contributed 14 new bonds, but the presence of the ‘long‘ linker resulted in a reduction to 28 bonds, likely by selecting the most favorable, strongest H-bonds. For ZNF-disrupted PHD extended with the ‘short‘ linker, H-bonding extent decreased to 50% (from 56 to 27).

The fourth parameter evaluated, the buried surface area (BSA), is used to measure binding site size and estimates binding affinity [19,20]. Average BSA per model spanned from ~1160 (PHD) to ~2140 (PHD+sl) Å^2^, which is the standard range for many stable protein-protein complexes with intermediate to large interfaces [21]. The largest BSA measured for PHD+sl is consistent with the high values of other parameters but is not necessarily proportionate to the increase in binding strength because, at BSA > 2000 Å^2^, the surface energy density levels off to a constant value [21]. Values for BSA are therefore presented as complementary and illustrative rather than as measures of binding affinity. Generally, high parameter values of both PHD+sl versions do not accurately reflect the real binding affinity because many contacts and interactions appear to be weak and/or transient. This is particularly pertinent to the large number of predicted vdW interactions (379 for wt and 331 for ZNFmut).

As stated above, it is evident from the experimental section (Figure 4) that the length of TIP5 protein fragments follows the sequence: PHD < PHD+sl < PHD+ll, in order of increasing histone octamer binding affinity. The highest binding affinity of PHD+ll, characterized by more vdW interactions (44 vs. 31) and fewer H-bonds (28 vs. 42), is a common phenomenon in molecular recognition. For example, it is applied in drug design and protein–ligand interactions, where increasing vdW interactions, by filling hydrophobic pockets, lead to stronger binding affinity [22].

Returning to histone H3, which is the previously reported main binding partner for TIP5 [4]; the middle section of Table 1 summarizes an analogous characterization of the binding properties of the histone octamer-complexed H3. Comparison with the entire octamer (bottom section) reveals that H3 *per se* is responsible for the majority of interatomic contacts, vdWs, H-bonds, and BSA with the PHD module. The percentage of H3 involvement in contacts and H-bonds gradually decreases with the presence and further extension of the linker. Notably, depending on linker length, there is a substantial difference in the H3 participation rate in the vdW interactions (~79% for sl vs. ~27% for ll). This suggests that the ‘long‘ linker shifts the distribution of vdWs to other octamer subunits. With regard to the disrupted zinc finger, H3 engagement in H-bonding decreases from 62.5% (PHD+sl) to ~33% (PHD+sl, ZNFmut), which is in accordance with the extensive hydrogen-bond interactions between the PHD zinc finger and H3 [4].

Attempting to interpret the significance of my experimental findings and computational structure predictions on the TIP5 association with the histone octamer, I discuss here the major roles of TIP5 in a cellular context. TIP5, as a subunit of chromatin remodeler NoRC, forms a molecular motor that acts on nucleosomes [23]. NoRC is involved in repositioning of nucleosomes at the ribosomal (r)DNA promoters, thus preventing the recruitment of RNA polymerase I (Pol I) machinery and ultimately silencing the rRNA genes [24,25]. NoRC also mediates the rDNA silencing by another mechanism, through promoting the heterochromatin formation at the Pol I promoters [26,27]. Further, NoRC controls the rDNA replication timing by shifting it from early to late S-phase, thereby establishing epigenetic repressive marks [28]. In healthy cells, TIP5 functions as a repressor of rDNA to maintain normal homeostasis, but TIP5 is often overexpressed in malignant cells, in which it promotes neoplasia and metastasis [29,30].

## 4. Conclusions

The ultimate goal of this study was to establish a foundation for our understanding of the association between the TIP5 protein and the whole histone octamer, as a fundamental scaffolding complex for DNA condensation. I selected the PHD module and PHD-BRD interdomain linker because these regions were previously reported to be involved, directly or indirectly, in the interactions with the post-translationally unmodified histone H3. A detailed examination of the exact role of the overly basic (Lys, Arg-rich) linker in association with the octamer is needed; however, the existence of a complex electrostatic interaction network is plausible. The basic linker residues may find partners within the “acidic patch” on the surface of the octamer. Conversely, the acidic residues (Asp, Glu) on the linker may interact with the positively charged histone residues. The initial experimental and structural basis provided here opens new avenues for investigation of the dynamic structural and functional interplay between chromatin remodelers and nucleosomes.

## Figures and Tables

**Table 1:** Key interaction parameters of the histone octamer (top section) and histone H3 (middle section) complexes: A comparison between the TIP5 PHD domain alone and three PHD-interdomain linker protein fragments. Number of contacts, van der Waals (vdW) interactions, and hydrogen (H)-bonds are calculated from a total five predicted models for each complex. Correspondingly, the sums of buried surface areas (BSA) were derived from five models and are given in square Å. The percentage of octamer-complexed H3 compared with the complete histone octamer is shown in the bottom section.

	Contacts	wdW	H-Bonds	BSA (Å^2^)
**Histone octamer**				
PHD	573	31	42	5814
PHD+sl	1692	379	56	10,679
PHD+ll	479	44	28	6225
PHD+sl, ZNFmut	1345	331	27	7154
**Histone H3**				
PHD	445	21	32	4167
PHD+sl	1186	301	35	5315
PHD+ll	307	12	15	3296
PHD+sl, ZNFmut	899	277	9	3047
**[%] of H3 from octamer**				
PHD	77.66	67.74	76.19	71.67
PHD+sl	70.09	79.42	62.50	49.77
PHD+ll	64.09	27.27	53.57	52.95
PHD+sl, ZNFmut	66.84	83.68	33.33	42.59

## Data Availability

The original contributions presented in this study are included in the article/Supplementary Material. Further inquiries can be directed to the corresponding author.

## Supplementary Materials

The following supporting information can be downloaded at: https://www.mdpi.com/article/10.3390/biom16081209/s1, Figure S1: AlphaFold 3 structural predictions of the histone octamer complex with two partial TIP5 protein copies. Figure S2: Original uncropped image of Figure 1A. Figure S3: Original uncropped image of Figure 1C. Figure S4: Original uncropped image of Figure 2A. Figure S5: Original uncropped image of Figure 4B. Figure S6: Original uncropped image of Figure 4C. Figure S7: Original uncropped image of Figure 5.
