# Supplementary material for: An Intact PHD Finger and PHD-BRD Interdomain Linker Are Crucial for Binding of the Chromatin Remodeler Factor TIP5 to the Histone Octamer
Source: Biomolecules. 2026 Aug 19;16(8):1209. doi: 10.3390/biom16081209 (PMC13510838; doi:10.3390/biom16081209)

SUPPLEMENTARY MATERIALS

An intact PHD finger and PHD-BRD interdomain linker are crucial for binding of the chromatin remodeler factor TIP5 to the histone octamer

Pavel Čabart

Department of Cell Nucleus Plasticity
Institute of Experimental Medicine of the Czech Academy of Sciences (IEM CAS)
Prague, 142 00
Czech Republic
correspondence: pavel.cabart@iem.cas.cz

CONTENTS

Page 2 1. Supplementary Figure S1 (related to Figure 6)
AlphaFold 3 structural predictions of the histone octamer complex with two partial TIP5 protein copies.

Page 3 2. Supplementary Figure S2
Original uncropped image of Figure 1A (SDS-PAGE).

Page 4 3. Supplementary Figure S3
Original uncropped image of Figure 1C (SDS-PAGE).

Page 5 4. Supplementary Figure S4
Original uncropped image of Figure 2A (SDS-PAGE).

Page 6 5. Supplementary Figure S5
Original uncropped image of Figure 4B (Western blot).

Page 7 6. Supplementary Figure S6
Original uncropped image of Figure 4C (Coomassie Blue-stained blot; related to Figure 4B).

Page 8 7. Supplementary Figure S7
Original uncropped image of Figure 5 (SDS-PAGE).

1. Supplementary Figure S1

Figure S1 refers to the main text (page 10, lines 331–336) of the manuscript.

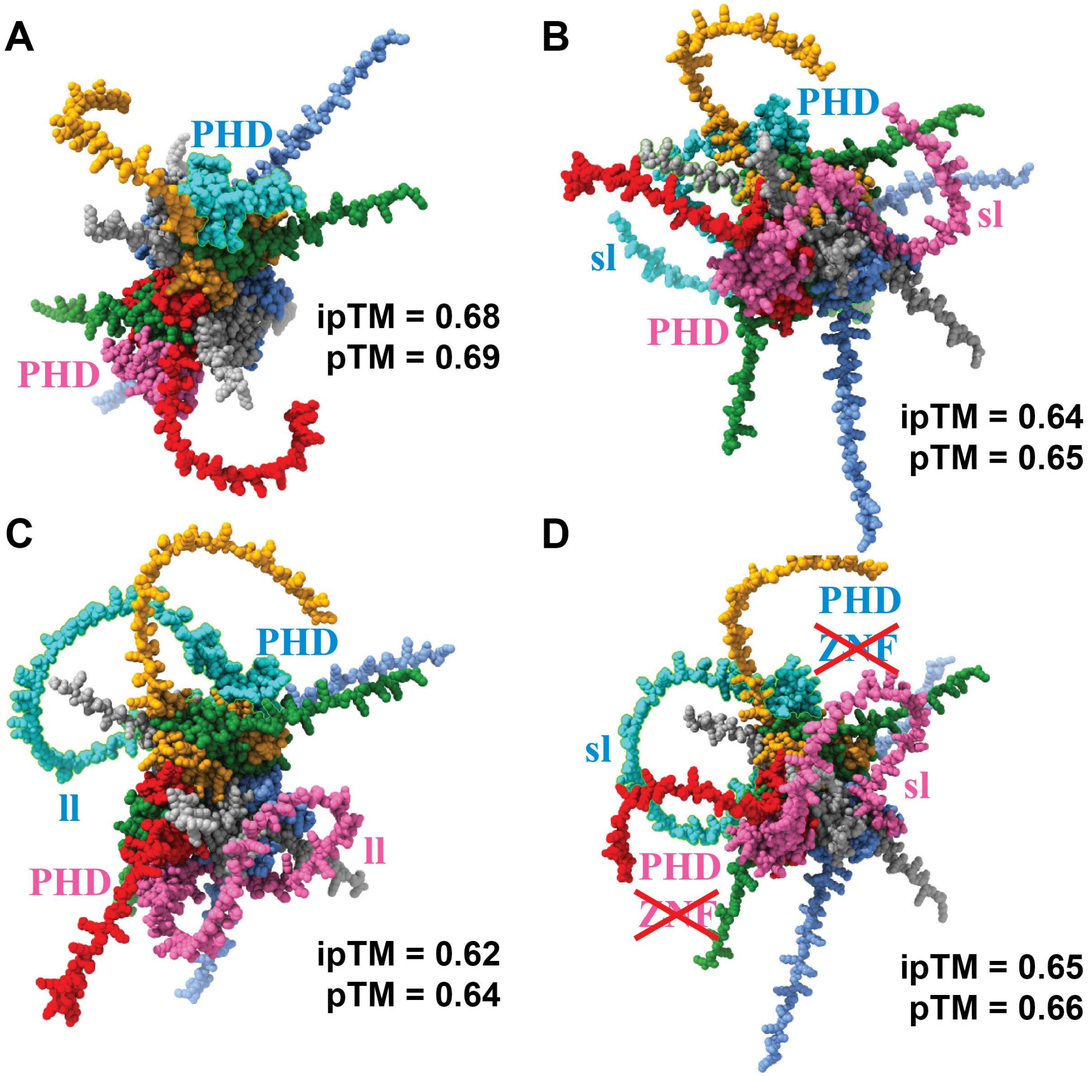

Figure S1 (related to Figure 6) Predicted 3D structures of two molecules of TIP5 PHD domain, and PHD extended by interdomain linker fragments, in complex with the octamer. AlphaFold 3 model of the **(A)** PHD domains alone (PHD), **(B)** PHD domains with the 'short' linker (PHD sl), **(C)** PHD domains with the 'long' linker (PHD ll), **(D)** PHD domains with the 'short' linker, zinc finger mutated (PHD sl ~~ZNF~~). All structures are portrayed in sphere style; one of the two copies of TIP5 partial proteins is displayed in cyan; the second copy of TIP5 partials in purple; H2A subunits in grey; H2B subunits in blue; one of the two copies of H3 subunit in orange; the second H3 copy in red; H4 subunits in green color. Structural confidence and accuracy metrics scores, pMT and ipMT, are shown for each model.

2. Supplementary Figure S2

Original uncropped image of Figure 1A (SDS-PAGE).

This image was used to generate Figure 1A on page 4 of the manuscript.

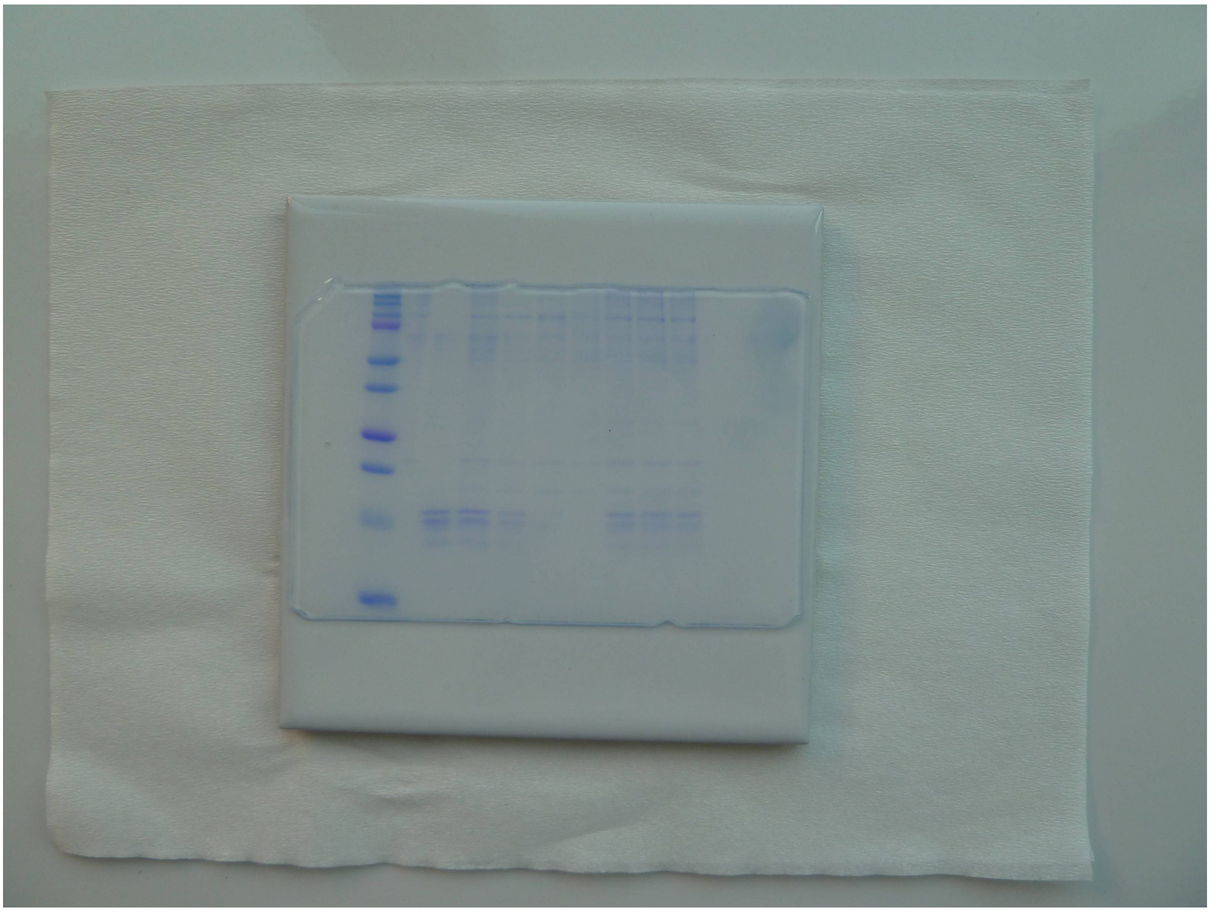

3. Supplementary Figure S3

Original uncropped image of Figure 1C (SDS-PAGE).

This image was used to generate Figure 1C on page 4 of the manuscript.

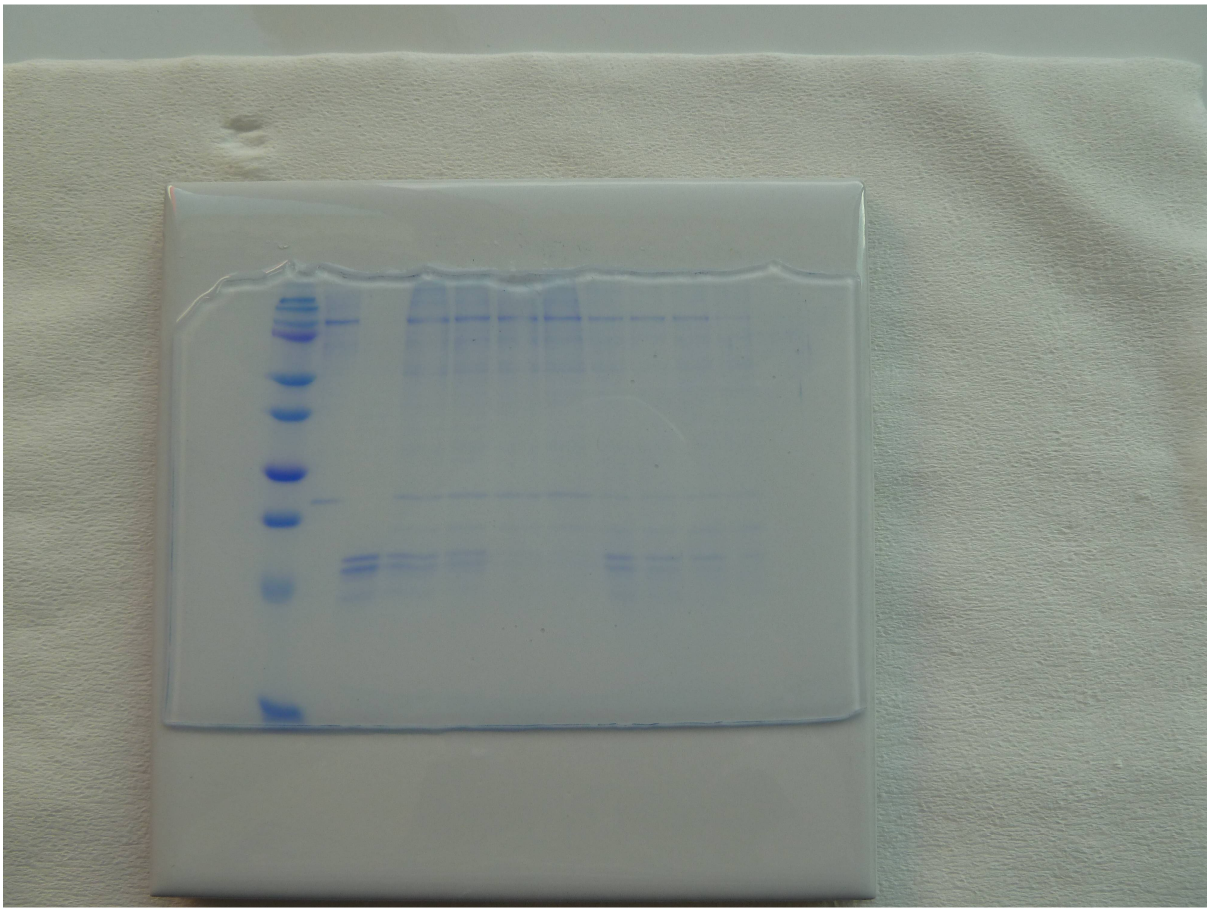

4. Supplementary Figure S4

Original uncropped image of Figure 2A (SDS-PAGE).

This image was used to generate Figure 2A on page 6 of the manuscript.

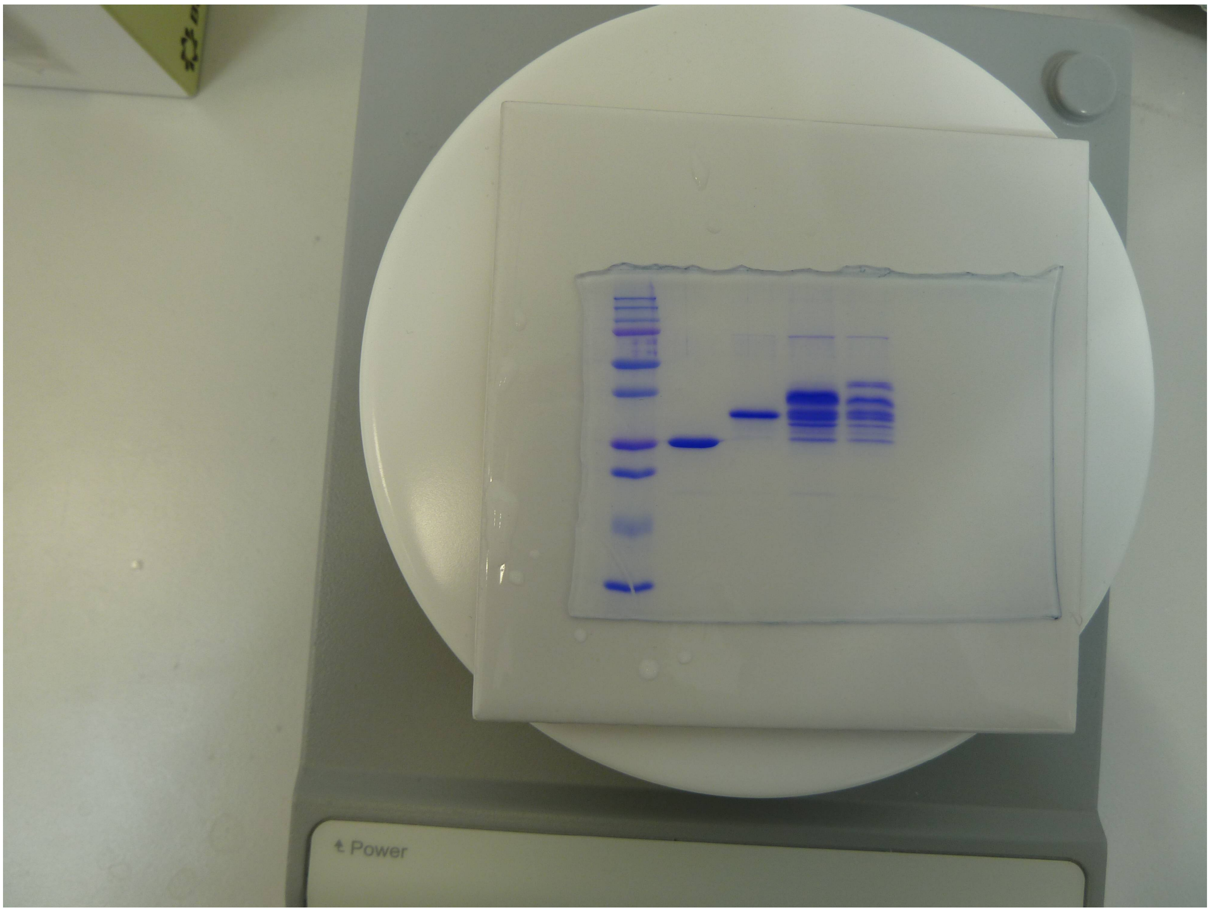

5. Supplementary Figure S5

Original uncropped image of Figure 4B (Western blot).

This image was used to generate Figure 4B on page 8 of the manuscript.

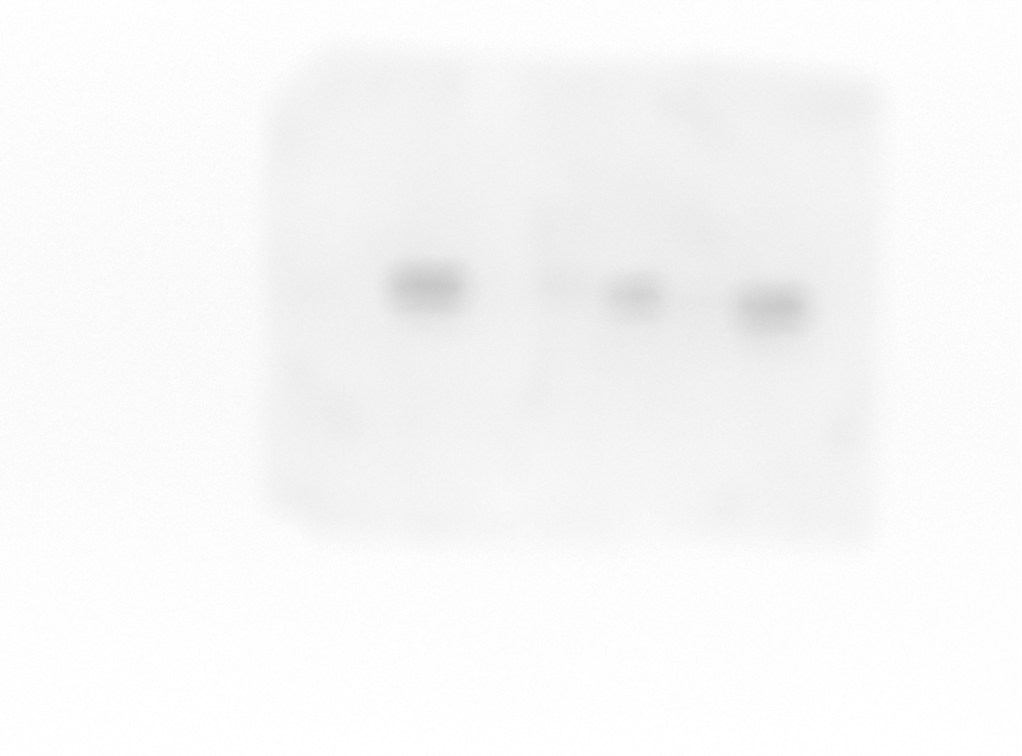

6. Supplementary Figure S6

Original uncropped image of Figure 4C (Coomassie Blue-stained blot; related to Figure 4B).

This image was used to generate Figure 4C on page 8 of the manuscript.

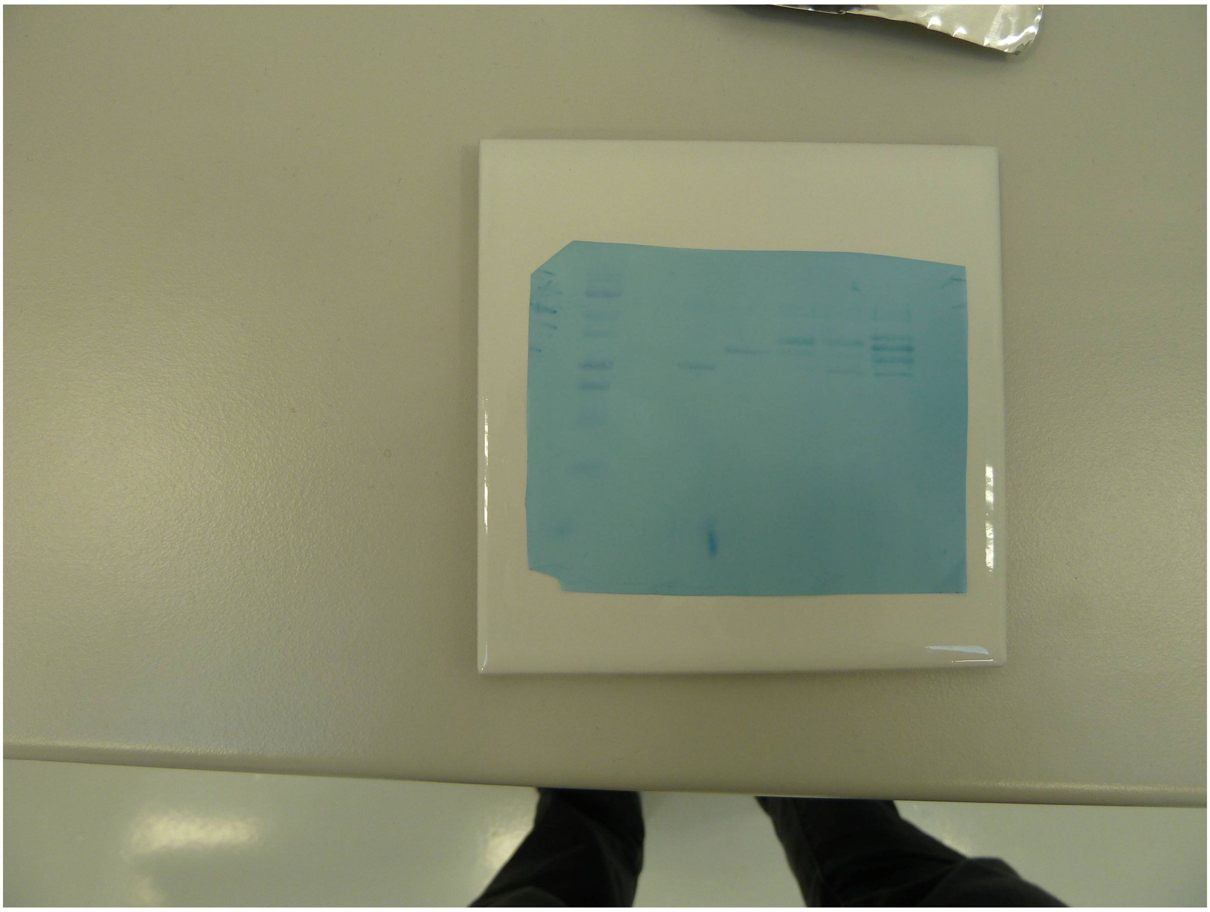

7. Supplementary Figure S7

Original uncropped image of Figure 5 (SDS-PAGE).

This image was used to generate Figure 5 on page 9 of the manuscript.

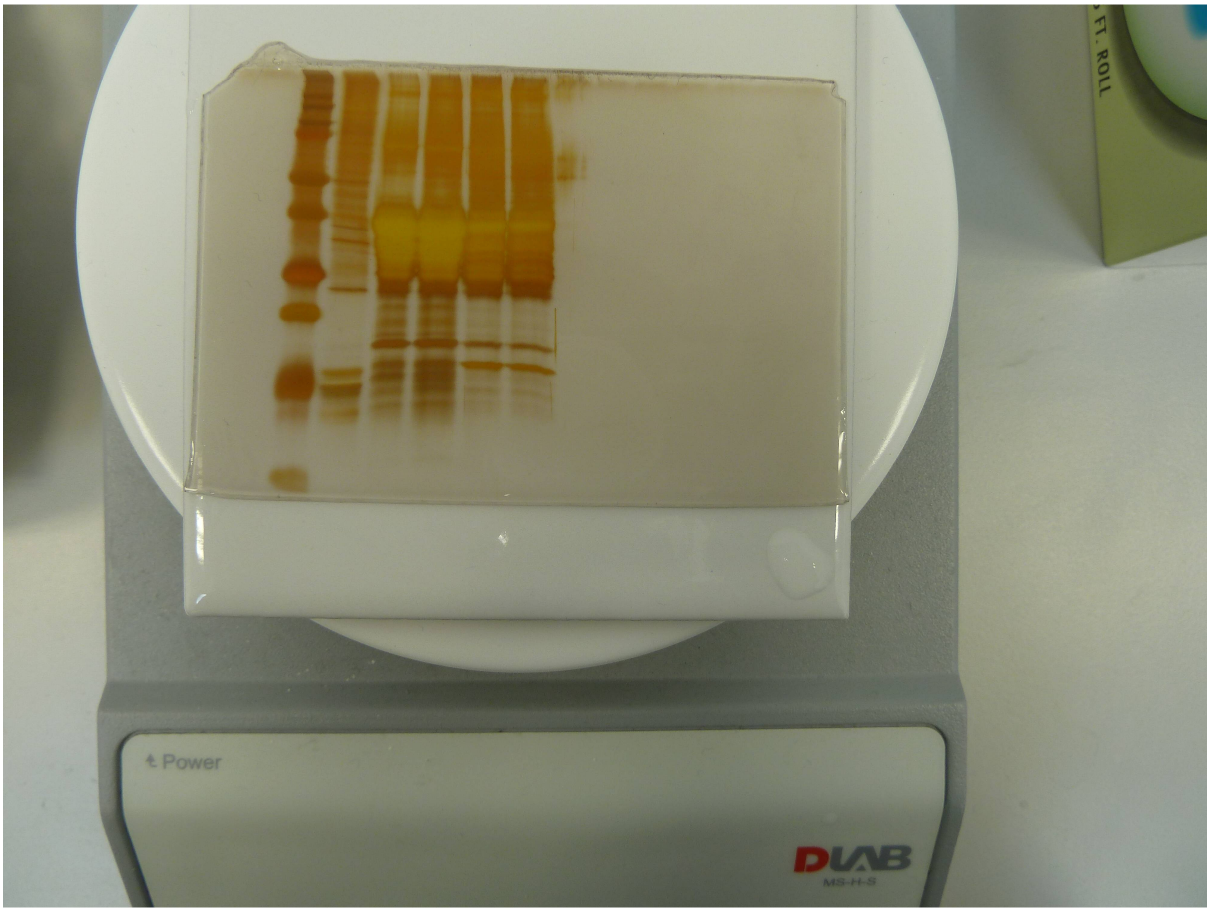

Supplement: Supplementary file 1 [file biomolecules-16-01209-s001.zip › biomolecules-4453309-supplementary.pdf]
